# Text messaging: an innovative method of data collection in medical research

**DOI:** 10.1186/1756-0500-3-342

**Published:** 2010-12-20

**Authors:** ST Kew

**Affiliations:** 1 Department of Internal Medicine, International Medical University, Kuala Lumpur, Malaysia

## Abstract

**Background:**

The ubiquitous use of mobile phones in sending and receiving text messages has become a norm for young people. Undeniably, text messaging has become a new and important communication medium not only in the social realm but in education as well. The aim of this study is to evaluate the effectiveness of using text messaging as a means to collect data for a medical research project.

A cross sectional study was carried out during a double blind, randomized controlled trial to assess the efficacy and safety of a probiotic in the management of Irritable Bowel Syndrome (IBS). The study aim was to assess the response rate of weekly symptom reports via Short Message Service (SMS). The subjects were undergraduates in a private medical university in Malaysia. They were identified through a previous university wide study as suffering from IBS based on Rome III criteria. The subjects were randomly assigned to either the treatment arm receiving a daily probiotic, or the placebo arm. They were required to score their symptoms using eight-item-questionnaires at baseline, and thereafter weekly, for a total of 8 weeks. All subjects were given the choice to communicate their symptom scores by text messaging via mobile phones or by email. SMS text messages were sent to remind trial subjects to attend face-to-face visits and to complete a paper based 34-item-questionnaires on IBS quality of life assessment at baseline and at end of 8 weeks.

**Findings:**

The response rate of weekly symptom scores via Short Message Service (SMS) from a total of 38 subjects was 100%. Through the study, 342 reports were submitted: 33.3% of these were received on the due date without reminder, 60.0% one day after the deadline, after a single reminder, 6.1% 2-3 days after the deadline, after 2-3 reminders and 0.6% 5 days after the deadline, after SMS, phone reminder and face-to-face encounter. All SMS symptom reports, whether on time or late, were complete. With the help of SMS reminder, all trial subjects completed the paper based IBS quality of life assessment at baseline and at end of study.

**Conclusions:**

This study found using text messaging via mobile phone an excellent instrument for collecting weekly symptom reports in response to trial medication, reminding trial subjects to attend face to face visits and completing more complex paper based evaluation. The 100% response rate of weekly symptom reports was facilitated by using simple number codes for SMS submission.

**Trial Registration:**

Not appropriate.

## Introduction

Mobile phones have revolutionalized our lives. Short message service (SMS), an integral part of the mobile phone system, has several attractions. SMS is cheap, user-friendly; messages can be stored, retrieved and answered at user's convenience; and transmission is as quick or almost as quick as any phone call. SMS is particularly popular with the young [1]. There is now a SMS culture, and even an emerging SMS language, the essence of which is basically "simple and short" as only up to 160 characters of text can be sent per transmission. It has been reported in a Malaysian daily (Star newspaper dated 12 May 2009 by columnist Abhinav Sharma) that young people, aged 16-24 years, send out anywhere between 40-60 text messages a day, and just about over 10 calls a day from their mobile phones. The mobile phone is considered an essential personal item by many young people, and is carried on the person almost any time of the day, at least during the waking hours. When asked how hard it would be to give up a specific technology, respondents are now more likely to say the cell phone would be the most difficult to do without, followed by the internet, TV, and landline telephone [1]. As a corollary, SMS via mobile phone has become a powerful and inexpensive tool in getting in touch with people. Because of their high rate of ownership and frequent use, mobile phones show great promise as a communication tool in every arena of modern living including health care.

Review of the medical literature found many and varied uses of SMS in the health care context. **In preventive medicine**, sending text message reminders on travel vaccine series completion was found to be an effective intervention measure, as compliance greatly improved with second and third doses of Hepatitis vaccines in the travel vaccine series [2]. Similar immunization reminders to parents of adolescent children were well accepted. Parents in general felt that they would act on these text messages to improve on-time vaccination for their adolescent children [3]. Both these papers detailed sending SMS reminders. The recipients were only required to retrieve and read the SMS on their mobile phone.

**In promoting healthy behaviours**: A randomized controlled trial found text messaging an effective method in improving adherence to sunscreen application [4]. The trial subjects were sent text messages but were not required to respond. In another randomized controlled trial, using SMS was effective in promoting weight loss over 4 months among a group of overweight and obese adults [5]. Besides receiving text messages, these subjects were required to report their weight once a week by SMS.

**In disease monitoring and self management**: text-messaging support system "Sweet Talk" enhanced self-efficacy, facilitated uptake of intensive insulin therapy and improved glycaemic control in paediatric patients with Type 1 diabetes mellitus [6]. Similarly in patients with asthma, a Danish study found SMS collection of asthma diary data feasible, and SMS was successfully used as a tool for supporting self-management of asthma [7]. Both these studies involved SMS interaction between study subjects and the researchers.

**In health behavior intervention**, e.g. smoking cessation: text messaging based smoking cessation programme (i.e. regular personalized text messages providing smoking cessation advice, support and distraction) about doubled the quit smoking rates in 6 weeks among young adults in New Zealand [8]. Likewise, a web and text messaging smoking cessation programme targeting college smokers found quit smoking rates comparable to most minimal contact or self-help smoking cessation interventions. Participants rated it high on acceptability, satisfaction and subjective ratings of success [9]. Both these studies also involved text message interaction between study subjects and the researchers: including sending SOS text message that indicated craving for cigarette, and need for urgent support.

There is growing interest in the possibilities of using SMS in **medical research**. In primary care research, the response rate of patients aged 16-24 years on their satisfaction with consultation was not significantly lower in the group using text messaging (n = 193, response rate 80.2%) compared to the group using card response method (n = 209, response rate 85.6%) [10].

A randomized controlled trial comparing SMS, paper and online diaries in collecting weekly sexual behavior found SMS to be convenient and timely, though online data collection was preferable and more likely to be complete. SMS was also considered more private than paper diary collection. Besides, SMS was more convenient as diaries can be completed and sent from anywhere and at any time [11].

Using text messaging to contact difficult to reach participants in "Safe Point" study, JE Maher et al reported that study participants found text messaging less of a time commitment and more private when others are present. Participants also felt less personal receiving text messages [12].

The aim of this study is to evaluate the effectiveness of mobile phone short message service (SMS) in collecting weekly symptom scores of trial subjects in a double blind, randomized controlled trial assessing the efficacy and safety of a probiotic in the management of Irritable Bowel Syndrome (IBS). Previous SMS studies did not look at ways to ease the burden of repeated submission of SMS responses in longitudinal research. In this study, the process of submitting weekly symptom report was simplified using number codes to facilitate response (Table 1 last column) [Additional file 1]. A complete response with all 8 items answered contained 33 numbers and punctuation marks in total [Additional file 1]. It was hypothesized that easing the burden of recording and submitting the weekly symptom report would improve response rate.

**Table 1:** Efficacy parameters assessed by weekly diary

*#*	*Efficacy Variable*	*Question*	*Response Scale*	*SMS code (choose one)*
1	Abdominal discomfort/pain	"How intense was your abdominal discomfort/pain in the past week?"	0 = none 1 = mild 2 = moderate 3 = severe	**1**-0 **1**-1 **1**-2 **1**-3

2	Bloating	"How intense was your abdominal bloating in the past week?"	0 = none 1 = mild 2 = moderate 3 = severe	**2**-0 **2**-1 **2**-2 **2**-3

3	Stool frequency	"On the average, how many bowel movements did you have per day in the past week?"	Number of bowel movements per day or per week: _______________/day or week (please delete as appropriate)	**3**-X/d or **3**-X/w

4	Stool consistency	"Please rate your average stool consistency in the past week"	According to Bristol stool chart 1 = separate hard lumps, like nuts 2 = sausage-shaped but lumpy 3 = like sausage or snake but with cracks on its surface 4 = like sausage or snake, smooth & soft 5 = soft blobs with clear cut edges 6 = fluffy pieces with ragged edges, a mushy stool 7 = watery, no solid pieces	**4**-1 **4**-2 **4**-3 **4**-4 **4**-5 **4**-6 **4**-7

5	Urgency	"Did you at any time in the past week experience urgency?" (having to rush to the toilet for a bowel movement?)	1 = yes, all the times 2 = yes, sometimes 3 = no	**5**-1 **5**-2 **5**-3

6	Straining	"Did you strain during or while trying to have a bowel movement in the past week?"	1 = yes, all the times 2 = yes, sometimes 3 = no	**6**-1 **6**-2 **6**-3

7	Sensation of complete evacuation	"Did you have a sensation of complete evacuation following your bowel movements in the past week?"	1 = yes, all the times 2 = yes, sometimes 3 = no	**7**-1 **7**-2 **7**-3

8	Overall satisfactory relief of IBS symptoms	"Over the past week, do you consider that you have had satisfactory relief from your symptoms of IBS?"	1 = Excellent relief 2 = Good relief 3 = Slight relief 4 = No relief 5 = Worse	**8**-1 **8**-2 **8**-3 **8**-4 **8**-5

### Subjects and methods

This is a cross sectional study conducted during a double blind randomized controlled trial comparing a probiotic with placebo in the management of Asian subjects with Irritable Bowel Syndrome (IBS). Ethics approval for the study was obtained from the Institutional Review Board (Reference number 135/2007, dated 11 April 2007). Subjects were identified to have IBS according to Rome III criteria in a previous university wide survey [13,14]. They were all undergraduate students in a private medical university. Those who had accepted the invitation to participate in this study and given informed consent were randomized into two groups: one group receiving the trial probiotic for eight weeks, and the other group receiving placebo. Both investigators and subjects were blinded to the treatment. A 2 week-run-in observation period preceded the 8-week-treatment period. Subjects were required to record baseline and weekly response to 8-item-questionnaires on the satisfactory relief of IBS symptoms, bowel function, ease of passage, and symptom score including abdominal pain, bloating, flatulence and urgency (table 1) through the entire 8 weeks. Subjects were also required to fill in a hard copy of the 34-item-IBS-QOL at the beginning, and at the end of the study [15,16]. The study consisted of 3 face-to-face visits, spaced at 4 weeks apart: second visit at 28 ± 2 days, and third visit at 56 ± 2 days. During these visits, weight, pulse and blood pressure were checked. In the second and third visits, information on any adverse drug reaction was also recorded.

The aim of this present study was to assess the response rate of weekly symptom reports via Short Message System (SMS). At the start of the study, trial subjects were given the option to communicate their weekly symptom scores either by text messaging via mobile phones, or by email. All of them chose the text messaging option. Baseline symptom scores were collected via SMS at the beginning of the study on a Monday. For subsequent 8 weeks on every Monday, trial subjects were instructed to submit symptom scores using SMS to the research assistant without prompting. The weekly symptom report using 8-item-questionnaires (table 1) was printed on the front and back of a thick pocket-sized card and given to individual trial subjects as a reminder to the different responses in each questionnaire. There were three to seven possible answers per item which the subjects could select from, based on their symptoms in response to trial medication. These answers were converted into simple number codes (table 1 last column) [Additional file 1]. The name and mobile number of the research assistant were printed at the bottom of the pocket sized card. When the research assistant did not receive the weekly response by Monday of the following week, a SMS reminder was sent on a daily basis for 3 consecutive days or till the arrival of the response, whichever being earlier. When the trial subjects failed to send in their weekly symptom report after 3 SMS reminders, they were reminded through phone calls. Subjects who were not contactable by phone were tracked down and met face to face. This was possible as this study was conducted in a single centre i.e. a private medical university with 2 campuses 70 km apart.

SMS reminders were also sent to trial subjects 2-3 days before the second and third face-to-face visits, at the end of 4 and 8 weeks respectively. At the third visit, the trial subjects completed the second set of paper based 34-item-IBS-QOL questionnaires.

Confidentiality was maintained as subjects were identified only by their respective mobile number, and the symptom data were communicated using number codes. Only the researcher, and the three research assistants had access to the identity of the trial subjects, as well as the symptom reports and other data submitted by them. Data analysis and reporting were anonymised.

## Results

The total number of subjects enrolled in the study was forty three (43). The final number who completed the 10-week-study was 38 (88.4%). Five subjects withdrew from the study: three of them had worsening of IBS symptoms, and another two discontinued for lack of efficacy. Nineteen (19) subjects were in the probiotic group, and the remaining nineteen (19) were in the placebo group. Mean age of the trial subjects was 22.0 ± 1.47 years. There were 20 males, and 18 female (M:F ratio of 1.1:1).

Each subject would submit his/her symptom scores at baseline and every week for 8 weeks. In total, 38 subjects would submit a sum of 38 × 9 = 342 symptom reports. All 342 reports were submitted via SMS, scoring 100% response rate. Of these, 33.3% were received on the following Monday without reminder, 60.0% were received a day later after a single reminder, and 6.1% 2-3 days later after 2-3 reminders. Two subjects were tracked down and met face-to-face on one occasion each when they failed to respond to 3 daily SMS reminders followed by phone calls. Both these two symptom reports were submitted by SMS 5 days after the due date, i.e. 0.6% of the total symptom report submission.

All SMS symptom reports, whether on time or late, were complete, with answer to every one of the 8 items. None of the trial subjects reported any difficulty using the simple codes to submit the symptom reports.

Trial subjects were required to complete the paper based IBS quality of life assessment at baseline and at end of study i.e. at the third face-to-face visit when weight, pulse and blood pressure were checked, and any adverse drug reaction recorded. A SMS reminder was sent 2-3 days before the appointment date for the face-to-face review. Again the response rate for IBS-QOL questionnaires was 100%. The 34-item questionnaires were answered completely by all trial subjects.

## Discussion

In this study, Short Message Service (SMS) was found to be a feasible method of collecting weekly symptom diary data from undergraduate students in a medical university. From the outset, we did not consider using paper diary for symptom scores. We were mindful that paper based diaries suffer from questionable validity, as reported by Stone et al [17]. In his study, patients with chronic pain reported high compliance with paper diaries, but actual compliance was low, and hoarding, in which diary cards were completed outside the defined time window, was common [17]. Lauritsen et al conducted a multi-centre, open and parallel trial involving patients with gastro-oesophageal reflux disease. The twice daily report of heartburn symptoms and loss of sleep by paper diaries (P-diaries) was compared with electronic diaries (E-diaries) and telephone diaries (T-diaries). The authors concluded that data in the P-diaries were not filled in at appropriate time, calling into question its reliability and validity. Whereas using E-diaries and T-diaries in this study improved accuracy and quality [18].

Previous studies using SMS text messaging for research data collection had been successful. DM Haller et al conducted a RCT in primary care research in which young users of primary care service was asked if they were satisfied with the consultation. There was a choice between sending in the replies by SMS, or by card to be completed before leaving the practice. A response rate of 80.2% for SMS replies was achieved compared to 85.6% for paper based card [10]. MSC Lim et al conducted a RCT comparing SMS, paper and online sexual behavior diaries. For 3 months, participants were required to report the number of sexual partners in the previous week, and details about each partner (i.e. regular or casual, new or previous partner, number of times having sex in the last week, use of condom). Of the three groups, 80.0% SMS diaries and 63.0% online diaries were submitted on the correct day. Of the paper diaries, 83.0% were completed on the correct date based on participants' self report, but were submitted to the researchers late. In this study, SMS were also considered more private than paper diaries collection, but more likely to be incomplete [11].

In terms of number of items to be reported weekly by trial subjects, our study was comparable to MSC Lim's study [11]. There were 8 item-questionnaires with between 3-7 possible answers per item that subjects had to select based on their symptoms in response to trial medication (table 1). The answers to these 8-item-questionnaires were converted to simple number codes (table 1 last column) [Additional file 1]. A complete response with all 8 items answered contained 33 numbers and punctuation marks in total [Additional file 1]. Our study scored a 100% response rate for symptom report submission via SMS. We believe simplifying the logistics by using simple number codes was an important factor in achieving this excellent response rate. Besides, all symptom reports submitted by codes were complete, with every item in the questionnaires answered. In terms of timeliness, 93.3% of weekly reports were submitted on time (33.3%) or one day late (60.0%). Thus we have proven our hypothesis that easing the burden of recording and submitting weekly symptom report via SMS was effective in achieving a good response rate.

It is important to note this 100% response rate for an otherwise tedious weekly routine. Though there was no direct comparison using conventional method of paper-based diary, this 100% response rate was very encouraging and very promising. Using SMS text messaging in collecting research data and simplifying the logistics of response submission open up possibilities for this technology to be used for more complex studies of longer duration in which more detailed SMS responses are required.

Nevertheless, there were several limitations to the study. The trial subjects, being undergraduates in a medical university, were better motivated and better informed than average young people. They probably also had a vested interest and knew the importance of compliance in ensuring the success of this study. They were also IT savvy, sending and receiving SMS was very much part of their lifestyle. Sample size was small (n = 38), trial subjects were followed up intensively, study was conducted in a single centre, making it relatively easy to manage. All three research assistants were fellow undergraduates awaiting transfer to partner universities. They shared the same lifestyles and knew the best ways of communication with the trial subjects. These factors have contributed in varying extent to the excellent response rate, and may not be generalisable to other settings.

The extent to which these results may be generalisable to an older population is perhaps unknown, but the growing expansion of text messaging far beyond the young suggests good potential for the application of this method in older age group as well [10]. According to Pew Internet Project survey 2007, in a typical day, 60% of "under age 30" adults sent or received text messages, compared to 32% in "30-49" age group, and 14% in "50-64" age group [1]. These numbers are likely to grow with time given the popularity of mobile phones, the convenience and the need for people to stay connected.

There were also challenges to the use of SMS. In this study, trial subjects had to bear the cost of their SMS responses. There were few instances where subjects' mobile phones ran out of credit, or ran out of battery, causing delay in response. Perhaps offering trial subjects mobile phone credits or prepaid phone cards may be a useful strategy to ensure compliance and limit potential bias related to cost.

## Conclusions

In this study to assess the role of a probiotic in the management of Asian subjects with IBS, using SMS as the only mode of weekly symptom scores collection was found to be very effective. This study scored a 100% response rate of weekly symptom report submission. The excellent result was facilitated by converting symptom scores to simple number codes making it easy for SMS submission. Paper-based IBS-QOL questionnaires similarly scored 100% response rate, facilitated by SMS reminders sent to trial subjects to attend face-to-face visits.

The advantages of SMS via mobile phone are many: it is cheap, can be sent from anywhere and at anytime, less intrusive and more private when others are present. Importantly it is the commonest mode of communication among the young [1]. In Malaysia, there is over 90% penetration of mobile phones, perhaps even higher penetration in urban centres. This high rate of mobile phone ownership opens up enormous opportunity for the medical fraternity to communicate and to reach out, in various aspects of health care as well as in medical research.

## Competing interests

The author declares that she has no competing interests.

## Authors' contributions

This paper was conceived and written by a single author ST.

## Supplementary Material

Additional file 1 **An example of weekly symptom reports via SMS**. A box depicting number and alphabet codes conveyed via SMS. Click here for file
