# Supplementary material for: Text messaging: an innovative method of data collection in medical research
Source: BMC Res Notes. 2010 Dec 20;3:342. doi: 10.1186/1756-0500-3-342 (PMC3022815; doi:10.1186/1756-0500-3-342)
Supplement: Additional file 1 — An example of weekly symptom reports via SMS. A box depicting number and alphabet codes conveyed via SMS. [file 1756-0500-3-342-S1.DOC]

**Additional file** 1

Title: An example of weekly symptom reports via SMS

Description: A box depicting number and alphabet codes conveyed via SMS

**Additional file 1: An example of weekly symptom reports via SMS**

**1**-1; **2**-1; **3**-2/d; **4**-4; **5**-3; **6**-3; **7**-1; **8**-2
